# Exploring the impact of brief training on student pharmacists' naloxone communication skills

**DOI:** 10.1016/j.pecinn.2023.100196

**Published:** 2023-08-03

**Authors:** Kelly Jankowski, Donna M. Evon, Amanda N. Stover, Trish Mashburn, Scott A. Davis, Delesha Carpenter

**Affiliations:** a UNC Eshelman School of Pharmacy, Chapel Hill, NC, USA; b Department of Public Health Sciences, Clemson University, Clemson, SC, USA; c Department of Medicine, The University of North Carolina at Chapel Hill, Chapel Hill, NC, USA; d Department of Pharmaceutical Outcomes and Policy, UNC Eshelman School of Pharmacy, Chapel Hill, NC, USA

**Keywords:** Naloxone, Communication, Student pharmacists, Patient education, Counseling

## Abstract

**Objective:**

To explore: a) whether videos that model naloxone communication skills improve student pharmacists' naloxone knowledge, self-efficacy and communication skills and b) whether outcomes differ between video versus written materials.

**Methods:**

Student pharmacists (*N* = 31) were randomized to either video or written materials training. Changes in naloxone dispensing barriers, self-efficacy, and naloxone knowledge were assessed via survey, while changes in naloxone communication were measured with a standardized patient assessment.

**Results:**

For the entire sample, knowledge and self-efficacy significantly increased and barriers to dispensing decreased. Communication improved significantly in both groups. In unadjusted analyses, students with video resources reported higher self-efficacy post-training. However, analyses that controlled for demographic characteristics and baseline measures found that training type did not significantly predict any outcome.

**Conclusion:**

Brief written or video-based naloxone training improved students' knowledge, self-efficacy, and communication. Given the small sample, results are inconclusive regarding impact of training material type on outcomes.

**Innovation:**

Teaching student pharmacists how to communicate about naloxone is important given increasing opioid overdose death rates. This study was innovative because it examined the impact of two training material types that can be delivered asynchronously and that pharmacy programs could incorporate into their curricula to improve students' naloxone communication skills.

## Highlights

• Naloxone is a life-saving medication used to reverse opioid overdoses.
• Community pharmacists are one of the most accessible health professionals.
• Pharmacists have unparalleled ability to provide naloxone education.
• Effective Naloxone counseling helps increase awareness and reduce overdose deaths.
• It is unknown which training method is helpful for student pharmacist naloxone skills.

## 1 Introduction

In the United States (U.S.), over 106,000 overdose deaths were reported in 2021, with 66% of those being opioid-related [1,2]. Naloxone is used to reverse opioid overdoses and is available in multiple formulations, including Narcan® nasal spray [3]. Although the overall naloxone dispensing rate has increased, the dispensing rate per high-dose opioid prescription is still low. According to the most recent update of the CDC pharmacy-based naloxone dispensing report, an estimated 9 million patients were dispensed a high-dose opioid prescription, and 406,203 were dispensed naloxone in 2018 [4].

Community pharmacists are one of the most accessible health professionals, with patients visiting community pharmacies significantly more often than primary care physicians (13 vs. 7 visits per year, respectively) [5]. Given their accessibility and monthly patient interactions, pharmacists have unparalleled ability to educate and dispense naloxone. However, in recent studies, community pharmacists and student pharmacists frequently omitted important naloxone counseling points [6,7]. Barriers to educating and dispensing naloxone include: lack of communication training, integrating counseling into workflow, and ethical concerns [8,9,10,11].

Effective naloxone training could increase pharmacists' confidence to communicate about naloxone [12,13]. However, it is unknown if written or video training resources are more effective at increasing self-efficacy [14,15]. Prior naloxone training studies have been limited to evaluation of longer trainings that may be more difficult to integrate into pharmacy school curricula [16,17,18,19,20,21]. These studies also did not directly compare the effectiveness of different naloxone training materials. Knowing which method is more effective would help pharmacy programs select impactful content for their curricula, especially updates on emerging topics, such as naloxone communication and opioid overdose. Additionally, as asynchronous and flipped classroom teaching formats are used more widely, it may be easier for students to learn via videos in a virtual format. Therefore, knowing if videos are an effective method for naloxone education may increase pharmacy programs' confidence in choosing this method for their learners. This exploratory analysis investigated whether brief video resources might improve student pharmacists' knowledge, self-efficacy and naloxone communication relative to written materials.

## 2 Methods

### 2.1 Setting

A convenience sample of student pharmacists was recruited from the University of North Carolina (UNC) Eshelman School of Pharmacy, which is a four-year professional program located in Chapel Hill, NC. Data were collected from November 2020 to April 2021.

### 2.2 Participant eligibility and recruitment

**Fig. 1:**
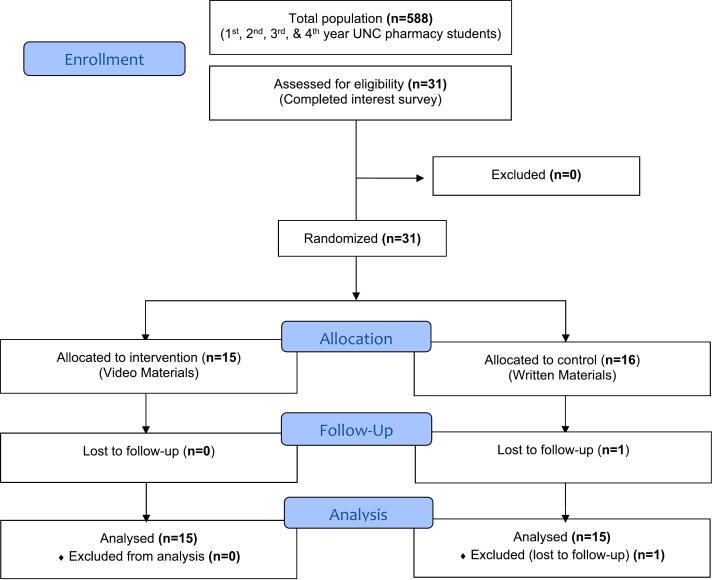
Enrollment.

All student pharmacists (first-year through fourth-year) were eligible to participate. Students who were part of the first author's research course were excluded from participation (Fig. 1). Participants were recruited via an email sent to the UNC pharmacy listserv and flyers shared with student organizations. Interested students were emailed the informed consent form.

### 2.3 Procedures

The UNC IRB deemed the study exempt (IRB #20–2327). Each student participated in two simulated patient (SP) scenarios: one pre-training and one post-training. Both SP scenarios were recorded on Zoom. The first author used a standardized script to simulate an individual consulting with a retail pharmacist. The scenario for both simulations involved a 32-year-old female with opioid and Narcan® prescriptions who visits a local pharmacy. The SP asked the student what Narcan® is used for and why her doctor would give her Narcan® since she has taken her pain medications for years and takes them exactly as prescribed. She also asked what she needs to know about it, how to use it, and what it costs.

This SP script was developed based on responses from 40 interviews with patients at high-risk of overdose [21]. A panel of community pharmacists and individuals with expertise in healthcare communication and substance use developed the script. Training to enact the script involved three training sessions where the first author practiced enacting the scenario with pharmacists on the research team in order to convey authenticity.

Following the initial encounter, students completed a baseline survey and were randomized to either video or written materials. Students randomized to the written material group were not given access to the video and vice versa. Students were given 2 months to complete the training due to competing course and experiential education demands. The second SP encounter and post-training survey was completed 1–2 weeks post-training. Students had up to 3 weeks to complete the surveys after encounters; a maximum of 3 weekly reminder emails were sent. Pre- and post-training communication was evaluated by two blinded coders using an observation guide and with reference to Zoom video recordings.

### 2.4 Randomization

Students were randomized with a random number generator to receive either written training materials (control; *N* = 16) or video training materials (experimental; *N* = 15).

### 2.5 Interventions

The intervention group received a 20-min “Narcan® Pharmacist Training” video and a 7-min “How to Use Narcan®” video from Narcan®’s website [3]. The first video included suggested verbiage for discussing counseling points and dispensing, while the second video showed proper administration technique. The control group received written handouts from naloxonesaves.org including 12 total pages of information on administration, counseling points, and counseling verbiage.

### 2.6 Sample size and statistical power

A sample size of 30 students was chosen due to feasibility, as the first author completed all 60 SP encounters [22]. Because this was an exploratory analysis, we examined effect sizes to explore potential trends in student outcomes based on group assignment.

### 2.7 Measures

Ten-minute surveys were completed online via Qualtrics.

#### 2.7.1 Barriers to dispensing naloxone

Students reported how concerned they were about 10 barriers to dispensing naloxone, including: lack of time and concerns it will make people use more opiates. Items were adapted from a previously validated questionnaire and additional questions were created based on a literature review on barriers to dispensing naloxone [23]. Responses were measured on a 4-point ordinal scale (1 = not a concern to 4 = major concern); higher mean scores indicated greater perceived dispensing barriers.

#### 2.7.2 Naloxone self-efficacy

Using a reliable and valid opioid overdose knowledge and attitudes measure [24], we adapted six items to measure students' self-efficacy to engage in various naloxone behaviors, including confidence to: (1) dispense to customers, (2) dispense according to NC state law, (3) educate customers to recognize opioid overdose signs, (4) use the teach-back method to assess patient understanding, (5) engage in counseling when the pharmacy is busy, and (6) discuss naloxone in a non-offensive way. Items were measured on a 4-point ordinal scale ranging from 1 = not at all confident to 4 = very confident.

#### 2.7.3 Naloxone knowledge

Seven knowledge questions were developed based on training content that was covered in both the control and experimental groups. Areas included what Narcan® is used for, how long Narcan® lasts, and administration techniques. Items were scored as incorrect = 0/correct = 1, with higher scores (range: 0–7) indicating higher levels of naloxone knowledge [24].

#### 2.7.4 Sociodemographic measures

Students reported age, gender identity, race, year in pharmacy school, and whether they had received any previous naloxone training.

#### 2.7.5 Communication measures

An observation guide based on a similar guide developed to assess the fidelity of an opioid overdose program was modified to assess students' naloxone communication during the simulated encounters (**see Supplementary Materials**) [25]. Observation guide items were modified according to prior literature and input from pharmacists and researchers on the study team [12,22]. This guide included several key counseling points that were presented in the training materials. Additionally, four verbal communication domains were rated and three non-verbal skills were assessed. The following scale was used: skill not demonstrated (1), skill needs development (2), or skill demonstrated with competence (3).

Four verbal communication domains were rated: (1) encouraged patient to have Narcan® in home, (2) thoroughly explained Narcan® administration, (3) spoke simply without using jargon, and (4) used clear/professional intonation of voice. Verbal communication scores could range from 3 to 12, with higher scores indicating better communication.

Additionally, three non-verbal communication skills were assessed: (1) expressed warmth, (2) used respectful demeanor, and (3) actively engaged with patient (made eye contact). The non-verbal scores could range from 3 to 9, with higher scores indicating better non-verbal communication.

Coders also documented: (1) whether students used an analogy (e.g., fire extinguisher) to describe naloxone (Yes, No, or Other), (2) the term used to describe an overdose (overdose, opioid overdose, opioid emergency, bad reaction, other), (3) whether they related the need for naloxone to patient-specific risk factors (e.g., presence of asthma), and (4) if they needed a “how do I use it?” prompt prior to discussing administration. A.

### 2.8 Data analysis

Two coders who did not interact with the students were trained to code naloxone communication with the observation guide. Coders were blinded to the group assignment and pre- vs. post-assessment. After introductory training, coders were provided with both sets of Narcan® training resources to gain a better understanding of what was covered and were given three example videos to code. Discrepancies were discussed and coders identified ways to increase inter-coder reliability. This process was repeated until the coders achieved >75% inter-coder reliability, at which time they began to separately code the remaining encounters.

Quantitative data were analyzed using SPSS Version 26 (Armonk, NY). Descriptive statistics were calculated and unadjusted pre-post differences in outcome variables (barriers, self-efficacy, knowledge, verbal communication, non-verbal communication) were examined using chi-square statistics and *t*-tests as appropriate. Linear regressions were then conducted with group assignment as the main independent variable. Regressions included the following co-variates: age, gender, race (white vs. non-white), year in pharmacy school, and previous naloxone training. In all regressions, the baseline measure of the outcome variable of interest was included as a control variable. For example, the regression for post-training self-efficacy controlled for baseline self-efficacy.

## 3 Results

### 3.1 Sample characteristics

The intervention group had significantly more females (*p* < 0.05) (Table 1). Six (19%) students had previous naloxone training, ranging from a recent topic discussion during a community rotation to a class-based skills session in a pharmacy school class.

**Table 1:** Baseline characteristics of intervention (video training materials) versus control (written training materials) group participants (*n* = 31).

	Intervention group n (%) (*N* = 15)	Control group n (%) (*N* = 16)	*p*-value
Demographics			
Age, years	24 ± 1.4	24 ± 2.2	0.34
Gender, female	15 (100)	11 (69)	< 0.05
Race, White	11 (73)	6 (38)	0.34
Race, African American	0 (0)	1 (6)	–
Race, Asian	3 (20)	7 (4)	–
Race, African Indian/American Native	0 (0)	1 (6)	–
Race, Other	1 (7)	1 (6)	–

Year in Pharmacy School			
PY1 (first year)	5 (33)	3 (19)	0.24
PY2 (second year)	4 (27)	2 (13)	–
PY3 (third year)	6 (40)	7 (44)	–
PY4 (fourth year)	0 (0)	4 (25)	–

Future Career Plans			
Ambulatory Care Pharmacy	3 (20)	2 (13)	1.00
Community Pharmacy	2 (13)	3 (19)	–
Hospital Pharmacy	7 (47)	8 (50)	–
Industry	1 (7)	1 (6)	–
Other	1 (7)	2 (13)	–

Previous Naloxone Training			
Yes	4 (27)	2 (13)	0.38

### 3.2 Barriers to dispensing

For the entire sample, barriers to dispensing naloxone significantly decreased after training; from 22.5 (SD 6.1) pre-training to 17.7 (SD 5.4) post-training, (t_(29)_ = 6.96, *p* < 0.001). In unadjusted analyses, there were no significant differences by intervention group (Table 2). Although intervention group was not significant in the regression model, two variables were significant: students who reported more barriers at baseline reported more barriers post-training (p < 0.001, B = 0.55, 95% CI (0.28, 0.82)), and students in third and fourth years of pharmacy school reported fewer barriers post-training compared to those in first and second-years (*p* < 0.05, B = -2.02, 95% CI (−4.03, −0.01)) **(**Table 3**)**.

**Table 2:** Descriptive statistics for intervention (video training materials) (N = 15) and control group (written training materials) (N = 15).

	Intervention Group (Video)		Control Group (Written Materials)		
Pre-Score	Mean ± SD	Range (min, max)	Mean ± SD	Range (min, max)	p-value*
Barriers to Dispensing	22.4 ± 7.2	26 (14, 40)	22.6 ± 1.2	15 (15, 30)	0.14
Self-efficacy	12.9 ± 4.9	16 (6, 22)	12.1 ± 2.7	10 (12,23)	0.06
Naloxone Knowledge	3.9 ± 1.4	5 (1, 6)	4.1 ± 1.2	4 (2, 6)	0.76
Verbal Communication	7.3 ± 1.7	6 (4, 10)	6.8 ± 1.3	5 (4, 9)	0.11
Non-Verbal Communication	6.1 ± 0.6	2 (5, 7)	6.1 ± 1	4 (5, 9)	0.30
Post-Score	Mean ± SD	Range (min, max)	Mean ± SD	Range (min, max)	p-value*
Barriers to Dispensing	19 ± 6.9	27 (12, 39)	16.5 ± 3.2	10 (12,22)	0.12
Self-efficacy	20.3 ± 2	8 (16, 24)	18.9 ± 3.4	11 (7, 17)	<0.05
Naloxone Knowledge	4.9 ± 1.2	4 (2, 6)	4.5 ± 0.9	3 (3, 6)	0.46
Verbal Communication	8.4 ± 1.3	5 (6, 11)	8.9 ± 1.6	5 (7, 12)	0.83
Non-Verbal Communication	7.1 ± 1.3	3 (6, 9)	6.9 ± 1.3	3 (6, 9)	0.34

Note: *p-value comparing intervention and control group without adjusting for co-variates.

**Table 3:** Linear regression model predicting barriers to dispensing naloxone, naloxone self-efficacy, naloxone knowledge, counseling points, verbal communication, and non-verbal communication after reviewing training materials (*N* = 30).

Variable	Barriers to dispensing naloxone Beta (95% CI)	Naloxone self-efficacy Beta (95% CI)	Naloxone knowledge Beta (95% CI)	Counseling points Beta (95% CI)	Verbal communication Beta (95% CI)	Non-verbal communication Beta (95% CI)
Intervention Group (Video)	−2.55 (−5.45, 0.36)	−0.89 (−3.03, 1.25)	−0.37 (−1.38, 0.64)	0.42 (−1.57, 2.40)	0.32 (−1.26, 1.91)	−0.08 (−1.32, 0.93)
Baseline	0.55 (0.28, 0.82)***	−0.26 (−0.59, 0.07)	0.26 (−0.19, 0.70)	0.30 (−0.26, 0.85)	0.33 (−0.16, 0.81)	0.64 (−0.001, 1.28)*
Age	−0.12 (−0.90, 0.66)	−0.23 (−0.82, 0.36)	−0.09 (−0.37, 0.18)	0.40 (−0.13, 0.92)	0.16 (−0.24, 0.57)	0.38 (0.09, 0.67)**
Gender (Female)	−1.00 (−4.78, 2.78)	−0.90 (−3.89, 2.08)	−0.88 (−2.22, 0.45)	−0.82 (−3.44, 1.80)	−0.28 (−2.27, 1.70)	0.45 (−0.94, 1.84)
White Race	−1.10 (−4.16, 1.97)	−0.89 (−2.94, 1.15)	−0.27 (−1.22, 0.68)	−0.25 (−2.10, 1.61)	0.31 (−1.19, 1.81)	0.03 (−1.08, 1.13)
Year in Pharmacy School	−2.02 (−4.03, −0.01)*	2.97 (1.45, 4.49)***	0.22 (−0.59, 1.03)	0.02 (−1.30, 1.33)	−0.24 (−1.21, 0.72)	−0.36 (−1.00, 0.29)
Previous Naloxone Training	1.19 (−2.10, 4.48)	0.81 (−1.70, 3.33)	−0.32 (−1.40, 0.76)	−0.76 (−2.86, 1.34)	−0.17 (−1.80, 1.46)	−0.20 (−1.34, 0.94)

Note: *p < 0.05 ** p < 0.01 *** p < 0.001; Barriers were measured using a scale of 1–4 with higher scores reflecting higher concern with dispensing naloxone. Self-efficacy was measured using a scale of 1–4 with high scores reflecting higher self-efficacy. Naloxone Knowledge was measured using a point system (0–7) with higher scores reflecting more knowledge about naloxone. Counseling points were measured using a point system (0–8) with higher scores reflecting more counseling points mentioned during simulated encounter. Verbal communication was measured using a scale of 3–12 with high scores reflecting higher competence with verbal communication. Non-verbal communication was measured using a scale of 3–9 with high scores reflecting higher competence with non-verbal communication.

### 3.3 Self-efficacy

For the entire sample, naloxone communication self-efficacy increased significantly from pre- to post-training; mean scores were 12.5 (SD 3.9) pre-training and 19.6 (SD 2.8) post-training, (t_(29)_ = 8.59, *p* < 0.001). In unadjusted analyses, the intervention group reported significantly higher self-efficacy post-training than the control group (*p* = 0.047) (Table 2). Intervention group was not significant in the regression model, but students in third and fourth years of pharmacy school reported higher confidence post-training compared to those in first or second years (p < 0.001, B = 2.97, 95% CI (1.45, 4.49)) **(**Table 3**)**.

### 3.4 Naloxone knowledge

Naloxone knowledge scores improved significantly from pre- to post-training; with mean knowledge scores of 3.97 (SD 1.3) pre-training and 4.70 (SD 1.1) post-training (t_29_ = 2.89, *p* = 0.007). In unadjusted analyses, the intervention and control group post scores were not significantly different (Table 2). No variables were significant in the regression model (Table 3).

### 3.5 Counseling points

The number of counseling points increased from 2.27 (SD 1.8) pre-training to 5.13 (SD 2.0) post-training (t_29_ = 6.83, *p* < 0.001). In unadjusted analyses, the intervention and control group post scores were not significantly different (Table 2). No variables were significant in the regression model (Table 3).

### 3.6 Non-verbal communication

For the entire sample, non-verbal communication improved significantly from pre- to post-training; mean non-verbal communication scores were 6.10 (SD 0.8) and 7.00 (SD 1.3), respectively (t_(29)_ = 4.38, p < 0.001). In unadjusted analyses, there were no significant differences by intervention group (Table 2). In the regression model, intervention group was not significant. However, students with lower baseline non-verbal communication scores had lower scores post-training (*p* < 0.05, 0.64 (−0.001, 1.28)) and older students had higher non-verbal communication post-training (*p* < 0.01, 0.38 (0.09, 0.67)) **(**Table 3**)**.

### 3.7 Verbal communication

For the entire sample, verbal communication and naloxone knowledge improved significantly from pre- to post-training. Specifically, mean verbal communication scores were 7.03 (SD 1.5) pre-training and 8.63 (SD 1.5) post-training (t_(29)_ = 5.00, *p* < 0.001). Naloxone knowledge scores improved from 3.97 (SD 1.3) pre-training to 4.70 (SD 1.1) post-training (t_(29)_ = 2.89, *p* = 0.007). In unadjusted analyses, there were no significant differences by intervention group (Table 2). Additionally, no variables were significant in the regression models (Table 3).

Students used different terms to describe naloxone's purpose post-training. Specifically, the term “overdose” was used less frequently (*n* = 12 pre and *n* = 5 post, *p* = 0.04). Additionally, the term “opioid overdose” (*n* = 9 pre and *n* = 7 post) was used less frequently post-training, though this decrease was not statistically significant. The terms “opioid emergency” (*n* = 0 pre and n = 5 post) and “bad reaction” (n = 0 pre and *n* = 3 post) were used more often post-training.

Training did not significantly increase the number of students who compared naloxone to a fire extinguisher or EpiPen (n = 5 pre-training and *n* = 6 post-training). However, several students compared naloxone to a seatbelt (*n* = 4) or a security blanket (n = 1) post-training; these terms were not used by any students before training. On the post-training survey, two students reported that the video module was too long (20:49) and monotone, making it difficult to watch in one sitting. Additionally, one student suggested that the written resource should include information on administration to children. Another student requested more information on the North Carolina standing order.

## 4 Discussion and conclusion

### 4.1 Discussion

This exploratory analysis investigated whether video or written naloxone training improved student pharmacists' knowledge and communication skills and if outcomes differed by training modality. The evidence was inconclusive as to whether video or written materials were more effective, with one exception that students who viewed videos reported significantly higher self-efficacy post-training. Although differences in outcomes between students appeared small, as a whole, the sample demonstrated significant improvements in knowledge, self-efficacy, and communication after completing brief written or video trainings. This finding is encouraging as it may be more feasible for pharmacy schools to integrate brief naloxone trainings into curricula, which could promote higher quality student counseling during pharmacy practice experiences. This is especially important since the Federal Drug Administration (FDA) recently approved a nasal spray formulation of naloxone (Narcan®) to be available over the counter. Therefore, pharmacists may receive more questions about naloxone.

Overall, students reported fewer barriers to dispensing after training. Students also felt significantly more confident post-training. Pharmacy students in higher class years reported fewer barriers and more confidence with counseling and dispensing naloxone. This finding is expected, given that student pharmacists who have had more opportunities to interact with patients should have greater confidence when counseling patients. This also suggests that students in earlier years may benefit most from brief naloxone training.

As a whole, students demonstrated improved verbal and non-verbal communication after training. Older students had higher non-verbal communication ratings post-training, suggesting that age plays a role in effectively communicating about naloxone, regardless of year in pharmacy school. Interestingly, there was not a significant increase in students using the term “fire extinguisher”, although it was mentioned in both resources. The term “seatbelt” was used significantly more after training, which shows that many students found the suggested analogy helpful when explaining naloxone's purpose. Less use of the word “overdose” post-training indicates that training can encourage students to use less stigmatizing verbiage [26].

This study has several limitations. This analysis was not powered to detect significant differences among trainings. Nonetheless, preliminary data were provided, including effect size data, to help design a future study. Generalizability is also limited, as the study included a small sample of volunteers at one university. In future studies, it would be helpful to not only assess ethnic differences, but also whether English as a first language or pharmacists' confidence with English, affect outcomes. Selection bias is a potential limitation as only students most interested in naloxone likely participated. Even though year in pharmacy training was not significantly different between the control and experimental group, there were more advanced (PY3 and PY4) students in the control group, which could have biased results to the null. Although the trainings did not contain identical information, we ensured that surveys and observation guide questions assessed topics that were addressed in both resources. We also could not verify whether students actually watched the videos. Additionally, it is possible that students could have searched for naloxone videos prior to their second SP encounter. Training time between completion and assessment on communication also varied between students. The SP was a pharmacy student, which could have influenced the interactions with study participants. However, blinded coders who did not know the participants coded the communication data.

### 4.2 Innovation

As opioid overdose death rates continue to rise, it is critical to equip student pharmacists with knowledge and communication skills so they can effectively communicate with patients about naloxone in practice. Because the FDA recently approved Narcan® an over-the-counter product, the number of patients consulting pharmacists in-person or over the phone with questions regarding naloxone use will likely increase.This further highlights the need for additional communication training options for student pharmacists.

The implementation and evaluation strategies used in this pilot study align with the specific framing for innovation in multiple ways. Our study developed a novel and innovative training method that pharmacy programs can use to improve naloxone knowledge and confidence to communicate about naloxone. The results could also influence training methods used by community pharmacies or hospital pharmacies to educate their pharmacists. Additionally, the novel research methods and surveys we used and developed could be used to support future research to evaluate different training interventions for pharmacy students and local pharmacists. These tools include the communication observational guide and new survey measures, such as the barriers to dispensing naloxone questionnaire and naloxone knowledge survey, to specifically fit the needs of evaluating naloxone. The SP script was also developed for this pilot study to simulate a scenario relating to naloxone and can be used in future studies. New research methods include the use of blinded coding of the recorded videos using the observational guide to assess communication skills.

This study also aligns with the general framing of innovation as its implications are related to novelty. Knowing that this topic is of increasing importance for pharmacy programs but that it is often difficult to create space in pharmacy curricula for additional topics, we evaluated the impact of two asynchronous teaching options (written and video materials). Using SP assessments, we found that both methods were effective at improving student communication about naloxone. This study is innovative because it is the first to specifically explore whether video-based naloxone communication training outperforms written materials for student pharmacists. Theoretically, videos should outperform written materials in improving communication self-efficacy since videos model behavior; this study provides the preliminary data needed to compare videos and written materials in an adequately powered future trial.

### 4.3 Conclusion

Brief naloxone communication training can improve student knowledge, self-efficacy, and communication. The associations between training type and study outcomes were inconclusive. These preliminary data will be useful in designing a future intervention trial with a larger, more heterogeneous sample to evaluate implementation outcomes and evaluate differences between training materials on student pharmacist outcomes.

## Funding support

This research did not receive any specific grant from funding agencies in the public, commercial, or not-for-profit sectors.

## Declaration of Competing Interest

The authors declare no relevant conflicts of interest or financial relationships.

## Appendix A Supplementary data

Supplementary Material Image 1
