# Supplementary material for: Exploring the impact of brief training on student pharmacists' naloxone communication skills
Source: PEC Innov. 2023 Aug 3;3:100196. doi: 10.1016/j.pecinn.2023.100196 (PMC10428026; doi:10.1016/j.pecinn.2023.100196)
Supplement: Supplementary file 1 — Supplementary Material [file mmc1.docx]

STUDENT ID NUMBER:

After all of the interviews are complete, the blinded raters will evaluate the student pharmacists’ communication skills

Who is completing this observational guide?

 Coder 1  Coder 2  other: ________

**Training Module Content:**

Compared Narcan to fire extinguisher or EpiPen?

 Yes  No  other: ________

Select All That Apply: Term used to describe what Narcan is for?

 overdose  opioid overdose  opioid emergency  bad reaction  other: ________

Related Narcan to patient-specific risk factors (personalized the discussion by relating need for Narcan to opioid scripts and/or presence of asthma)?

 Yes  No

Described at least (2) signs of drug overdose such as slow or stopped breathing, small “pinpoint” pupils, unarousable, limp body, pale/clammy skin, blue or purple lips/fingernails, or vomiting/gurgling noises.

 Yes  No

Was misinformation provided when discussing signs of opioid overdose?

 Yes ___________  No

**Proper explanation of administration technique:**

Needed the “How do I use it?” prompt prior to discussing administration?

 Yes  No

Made clear that patient cannot self-administer Narcan (explains that a caregiver will need to administer medication if patient has opioid overdose)?

 Yes  No

Mentioned moving the person on their side (recovery position)?

 Yes  No

Recommendation to call 911/emergency services?

 Yes  No

Informed patient that each box includes two separate doses of Narcan?

 Yes  No

Described when to use second dose if needed (says any time between 2-10 minutes, couple minutes, few minutes)?

 Yes  No

Acceptable description of cost for insured patients (explains it is typically covered by insurance for a small copay and/or student pharmacist can bill insurance and get back to patient with a price)?

 Yes  No

Acceptable description of cost for uninsured or cash paying patients (explains cash price is typically around $50-$150, coupons/programs are available to help patients pay for Narcan, and/or mentions standing order to help provide patients with Narcan)?

 Yes  No  N/A if identified patient has insurance (Medicaid)

**Verbal communication skills:**

| **Please rate with the following aspects of the Narcan encounter you observed** | **Skill not demonstrated**  **(0)** | **Skill needs development**  **(1)** | **Skill demonstrated with competence**  **(2)** |
| --- | --- | --- | --- |
| Student Pharmacist appears to encourage patient to have Narcan in home (i.e. makes an effort, still encourages after resistance) |  |  |  |
| Thorough explanation of administration of Narcan based on training resources |  |  |  |
| Student Pharmacist spoke simply and confidently without using jargon |  |  |  |
| Student Pharmacist has clear/professional intonation of voice |  |  |  |

Was misinformation provided during explanation of administration of Narcan?

 Yes ___________  No

**Non-verbal communication skills:**

| **Please rate with the following aspects of the Narcan encounter you observed** | **Skill not demonstrated**  **(0)** | **Skill needs development**  **(1)** | **Skill demonstrated with competence**  **(2)** |
| --- | --- | --- | --- |
| Student Pharmacist expressed warmth by making an effort to grasp the patient’s perspective and experience |  |  |  |
| Student Pharmacist used respectful demeanor with patient |  |  |  |
| Student Pharmacist was actively engaged with patient without reading off of notes (made an effort to maintain eye contact) |  |  |  |

Length of encounter: ______

Comments: ________
